# A Comprehensive Review of Canine and Feline Ventricular Septal Defects—From Pathogenesis to Long-Term Follow-Up

**DOI:** 10.3390/ani15060850

**Published:** 2025-03-15

**Authors:** Szymon Graczyk, Arkadiusz Grzeczka, Urszula Pasławska

**Affiliations:** Department of Diagnostics and Clinical Sciences, Institute of Veterinary Medicine, Faculty of Biological and Veterinary Sciences, Nicolaus Copernicus University in Torun, 87-100 Torun, Poland; grzeczka@umk.pl

**Keywords:** congenital heart defects, echocardiography, conotruncal defects

## Abstract

Congenital heart defects (CHDs) in dogs and cats represent a definite minority of cardiac patients. One of the most commonly diagnosed is ventricular septal defects (VSDs). These are associated with abnormal ventricular septation during the prenatal period; however, the mutations of the genes responsible for this phenomenon are not fully understood. VSDs pose a significant diagnostic challenge due to the multitude of locations in the ventricular septum where they are likely to occur. Therefore, there are many phenotypes of the defect causing many problems in terms of a common nomenclature. Among the various classifications, the latest terminology issued by The International Society for Nomenclature of Paediatric and Congenital Heart Disease (ISNPCHD) considers both electrical conduction pathways and adjacent structures to standardize nomenclature. Further, defects located at different sites can alter both prognosis and subsequent management for the patient; thus, taking accurate measurements is crucial. Among these, the Qp:Qs and VSD:Ao ratios, the direction of blood flow through the defect, its location, the diameter and maximum flow velocity, and the pressure difference between the RV and LV are indicated. Emerging technologies such as 3D echocardiography and cardiac magnetic resonance may provide additional diagnostic value. Altogether, along with clinical symptoms, we should determine further management, involving the monitoring of the patient, the implementation of pharmacological treatment, or referral for surgical closure of the VSD. This review summarizes current knowledge on VSD, where the pathogenesis of the condition, diagnosis, and management, including conventional and surgical methods as well as long-term follow-up, are described, providing a complete overview of the issue.

## Simple Summary

Ventricular septal defect (VSD) is a congenital heart disease characterized by an abnormal connection between the left and right ventricles. In dogs, it is the fourth most common congenital heart defect, while in cats it is reported most commonly. Depending on the size of the defect and its hemodynamic significance, it can remain asymptomatic throughout the animal’s life, or lead to significant cardiac remodeling and failure. The key aspect in the management of this type of defect is the constant cardiac monitoring of the patient, pharmacotherapy, and, as a last resort, surgery. Most VSDs are associated with a very good prognosis for the future in both dogs and cats.

## 1. Introduction

The emergence of a pathological orifice that allows communication between the left ventricle (LV) and right ventricle (RV) of the heart in the postnatal period is referred to as the ventricular septal defect (VSD). It is a condition classified as a congenital heart defect (CHD), with a pathogenesis not yet fully understood in humans, and even less so in dogs and cats. Admittedly, several genes whose mutations contribute to the development of VSD have already been revealed [1]. These studies have been directed mainly at humans; therefore, in the case of companion animals, this knowledge remains in the realm of hypothesis and conjecture. Moreover, it has been proven that one of the main genes responsible for the formation of VSD and atrial septal defect (ASD) in humans does not contribute to the defect in Doberman pinschers [2], highlighting the fact that genes attributed to VSD in humans may not be reflected in dogs and cats. Nevertheless, inheritance patterns of genes responsible for VSD formation have been proposed, but their exact identification has not been determined [3]. A certain difficulty among cardiologists in human, as well as veterinary, medicine is the lack of a standardized VSD nomenclature, and due to the multitude of VSD subdivisions available, the description of a defect is often a subjective assessment. For this reason, recently published guidelines on VSD nomenclature clearly outline the options for describing the defect by dividing it into four major classes and smaller subtypes [4]. Nevertheless, VSD should be considered an important and dangerous CHD, being the first in terms of prevalence in both humans [5] and cats [6], with a slightly lower prevalence in dogs, ranking it fourth among all CHDs in this species [7]. It is worth noting that VSD in about 50% of cases occurs in combination with other CHD, thus forming a complex defect. Therefore, the severity of the case will depend on the number and nature of the lesions, as well as coexisting CHDs. Depending on these lesions, different patterns of clinical symptoms can be observed, but most often patients are asymptomatic, with a predisposition for the disease to manifest itself later in life [8]. Based on the relevant parameters determining the hemodynamic significance of the changes due to VSD, some steps are taken regarding the further management of the patient. In most cases, treatment is not implemented due to the absence of significant cardiac morphological changes without cardiovascular consequences. However, there are cases with acute clinical symptoms, which force attending physicians, in addition to making a diagnosis, to assess the significance of the changes occurring and the possible decision to close the defect. The examination mode of choice is echocardiography, which is often supported by additional investigations, in particular X-ray and electrocardiography (ECG). Patients with significant hemodynamic changes should be referred for surgery; however, it should be remembered that the surgical closure of VSD in veterinary medicine is still not commonly performed. This is mainly related to the high cost of the equipment needed, the experience of the operators, as well as the financial capacity of the owners. However, there are currently reports of procedures performed using various techniques for VSD closure, including open heart surgery [9,10,11], the percutaneous closure of the defect using various occluders [12,13,14,15,16,17], or combining these two techniques and performing hybrid surgery [18]. Palliative procedures referred to as pulmonary artery banding, dedicated mainly to cats due to their smaller size and higher risk of postoperative complications associated with the placement of the occluder, are also discussed [19,20,21].

As a rule, an inconspicuous defect such as VSD can cause diagnostic problems. Its management is crucial to determine further steps and the possibility of the surgical correction of the defect, which may become increasingly common in veterinary medicine shortly. Therefore, the purpose of this review will be to provide a comprehensive discussion of VSD in dogs and cats, including pathogenesis, hereditary pathways, clinical signs, management, and the long-term follow-up of defects treated with conventional and surgical methods.

## 2. Cardiac Morphogenesis and Abnormal Development of the Interventricular Septum

**Figure 1:**
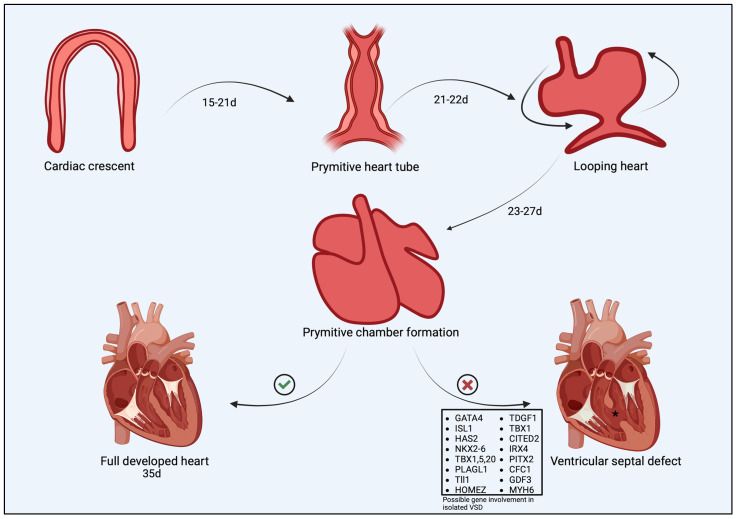
Cardiogenesis representing the 5 major stages of the forming heart, which include the cardiac crescent, the primitive heart tube, the looping heart, primitive chamber formation, and cardiac maturation. The box shows the possible contributions of genes responsible for isolated VSD, as derived from human medicine. d, day; VSD, ventricular septal defect.

The mechanisms that regulate normal cardiac development in humans have been studied for decades, but still, some processes are not fully understood. Much of this is attributed to cardiac development in dogs and cats, because cardiogenesis in mammals is based on similar mechanisms [22,23]. During the prenatal period, it is divided into four stages, starting with the formation of the primitive heart tube. After the descent of the mesodermal germ layer into the future cardiac field, a complex process of the induction of cardiac progenitor cell expression occurs through cardiac precursors SMARCD3 and cardiogenic transcription factors including gata family, Isl1, Tbx5 and Nkx2-5, which in turn enable the transcription of genes related to the programming of subsequent stages of heart formation [24,25,26]. Between 5 and 6 weeks, the first contractions associated with the action of the primary heart tube consisting of the areas of the sinus venosus, future atria, and ventricles, conus over future truncus arteriosus occurs [27]. Subsequently, through a mechanism called cardiac looping, the future shape of the heart is formed. The primitive heart tube typically twists to the right and folds into an S shape, where rapidly proliferating cardiomyocytes give rise to the heart cavities, as well as their outflow tracts [28]. The process by which the future heart differentiates into the atrial and ventricular myocardium along the outer curvature of the primitive heart tube has been called the ballooning model [26,29]. The atrial septum, which forms successively, and the interventricular septum are crucial for the proper development of the heart due to the separation of the systemic circulation from the pulmonary circulation. The development of the interatrial septum occurs via the overbuilding of cardiomyocytes derived from the endocardium-derived mesenchymal cap, AV cushions, and dorsal mesenchymal protrusion, where defects associated with the latter contribute to the formation of the atrioventricular septal defect (AVSD) [30]. The normal development of the interventricular septum involves the aggregation and coaptation of myocyte trabeculae until a full muscular septum is formed. Abnormalities associated with this process can result in muscular VSD. It is also crucial that septation terminates at the level of the AV valves, where the septum is thinnest and formed from the membranous portion, predisposing the individual to the perimembranous VSD that is most commonly documented in humans, dogs and cats [8,31,32]. Septation ends with the separation of the great artery outflow tracts and the complete division of the pulmonary circulation from the systemic circulation. These mechanisms are regulated by a well-defined cascade of gene expression [33], failures of which can end in CHDs such as VSD, ASD, or AVSD. Due to the lack of data in the canine and feline literature regarding the genetic pathways involved in VSD pathogenesis, it is difficult to determine which exact gene components contribute to this condition. On the other hand, many studies are available on the possible etiology of VSD in humans using mouse fetuses. To date, it is known that a large group of genes are involved in the etiopathogenesis of VSD, but the complexity and severity of the defect depends on the contribution of a particular gene. An abbreviated diagram of cardiogenesis and the contributions of possible individual genes to the pathogenesis of isolated VSD can be found in Figure 1 [1,34,35,36,37,38,39,40,41,42,43,44,45,46,47,48,49,50,51,52,53,54,55,56]. To date, indications of a much larger contribution of genes and their loci than previously thought point to the complexity of this defect. Due to the different contribution of possible genes in isolated VSD, VSD coexisting with other congenital heart defects, and VSD coexisting with other congenital defects outside the cardiovascular system, these defects were divided into isolated VSD, non-syndromic VSD, and syndromic VSD [57]. Each of these was associated with multiple mutations at the loci of multiple genes. Despite the intensification of CHD research, there are still many pathways that have not yet been fully explored, including primarily polygenic and oligenic heritability, the influence of environmental as well as genetic factors, non-coding variants of CHD, somatic mutations, and epigenetic factors.

### Mechanisms Underlying VSD in Dogs and Cats

**Figure 2:**
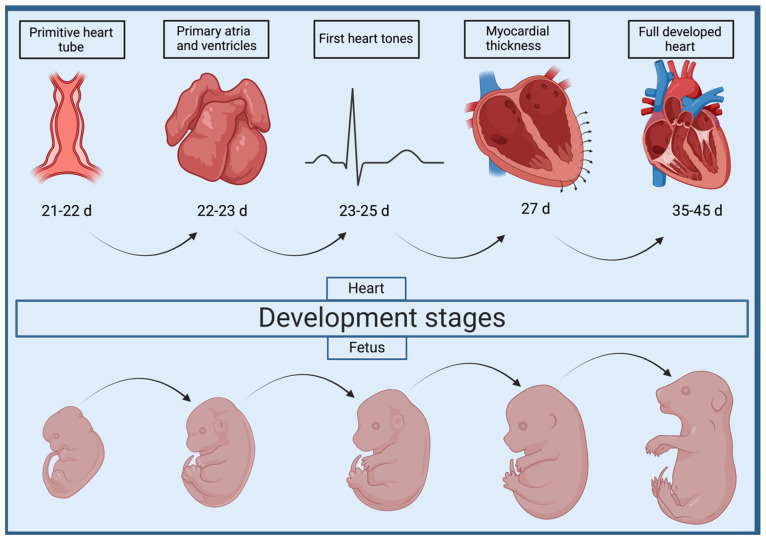
Scheme depicting the timeline of cardiac development in dogs and cats. The upper part of the figure shows consecutive changes in heart morphology and function. In the bottom part of the figure are the corresponding images of the developing fetus on a given day.

Due to the much shorter gestation period in dogs and cats, and thus their intensive organogenesis, cardiac development is considerably faster there than in humans. Namely, the primitive heart tube has been observed as early as day 21–22 of gestation, where from day 22 in dogs and day 23 in cats, the development of the heart septa and the formation of the primary ventricles and atria occurs [58]. The presence of four heart cavities in the form of two atria and two ventricles with a noticeable difference in myocardium thickness is noted by day 27 of gestation [59]. The first cardiac tones are observed during the formation of the heart chambers, i.e., between 23 and 25 days of age [60,61]. However, the full morphogenetic development of the internal organs, including the heart, ends around day 35, when the embryo becomes a fetus, and by the end of gestation, primarily exterior features develop [62] (Figure 2). In addition, on day 46, the histological image of the ventricular musculature is fully developed, with a significant predominance of the number and thickness of ventricular cardiomyocytes over the atria, especially the LV, which corresponds to the postnatal period [59]. No available studies show a link between VSD and a particular group of genes generating the defect in veterinary medicine. However, some papers have identified their inheritance patterns. In previous reports, researchers focusing their attention on VSD in dogs have also indicated a polygenic pathway of gene inheritance [63,64]. In addition, they assume that the higher the proportion of mutated genes, the more pronounced the severity and multiplicity of defects. The first stage has been defined as benign morphological changes in the heart, not revealing clinical symptoms. These have been distinguished as an absent papillary muscle of the conus, the persistence of the conus septal fusion line, or aneurysm of the ventricular septum [63]. Aneurysms are usually located in the membranous part of the septum. They may result from a spontaneously resoluted, hemodynamically insignificant VSD [65], or be a predisposing factor for aneurysm perforation and VSD formation, e.g., due to infective endocarditis [66,67]. The second stage already focuses on a more complex defect, which can contribute greatly to the deterioration of cardiac hemodynamics. Among these, VSD and pulmonic stenosis (PS) have been distinguished. The third stage includes CHD occurring in the same heart including VSD and PS, overriding aorta, atresia of the pulmonary valve, and even hypoplasia of the pulmonic trunk [63]. These data suggest that the occurrence of VSD and concomitant CHD depends on the involvement of various genes whose expressions are disrupted during cardiogenesis, and their synergistic negative effects result in a more complex defect. In addition, a group of genes (rather than a single gene) is also responsible for the occurrence of VSD, which confirms the fact that this defect can occur at different levels of the interventricular septum [64]. However, the same authors indicate that defects associated with conotruncal defect (CTD) are limited to mutations of a single gene with an autosomal recessive mode of inheritance, and the severity of CTD depends on the defect of this gene at different loci [68]. This hypothesis was challenged by Werner et al. (2005) [3], wherein the authors performed a genome-wide scan for CTD-linked loci in a Keshond x beagle family, and pointed to an oligogenic path of inheritance of this defect. The combined effects of three loci, occurring at least in pairs, contribute to abnormal conotruncal development. Notable among these are CFA2, CFA9 and CFA 15 [3]. Interestingly, in a study conducted on a beagle family, cross-infected offspring produced another generation with a VSD prevalence of 100%, which supports a model of autosomal recessive inheritance [69]. On the other hand, in the English springer spaniel family, autosomal dominant inheritance has been proposed as probably part of polygenic gene transfer [70]. Therefore, the inheritance of specific genes determining VSD should be considered in a complex and intricate manner, most likely involving several inheritance pathways, depending on specific genes.

## 3. Nomenclature of Ventricular Septal Defects

Over the years, various models for the nomenclature of VSD in human medicine have been defined, which have been drawn into veterinary medicine. However, due to the multiplicity of classifications and the subjective interpretation of the investigators, there has been a lack of consensus on a standardized nomenclature. The localization of the defect has typically been based on two basic principles, wherein each emerging guideline used modifications of previously mentioned classifications. These were based on a division according to the geographic nature of the defect, defining its location relative to the interventricular septum as seen from the lumen of the RV. This division seemed to be a suitable approach because of the characteristic topography of the anatomical structures and the shape of the RV [71]. This concept has allowed surgeons to determine the best surgical approach to close the defect. The second method of nomenclature is based on the physiological–anatomical borders of the defect, allowing the visualization of the electrical conduction pathways of the heart to avoid heart block during surgery [72]. A historical description of the different subtypes of VSD can be found in Table 1. Newer classifications are based on the consideration of physiological as well as topographic descriptions, so in 2000, The Society of Thoracic Surgeons proposed a naming system based on four main types—subarterial, perimembranous, inlet and muscular [73].

**Figure 3:**
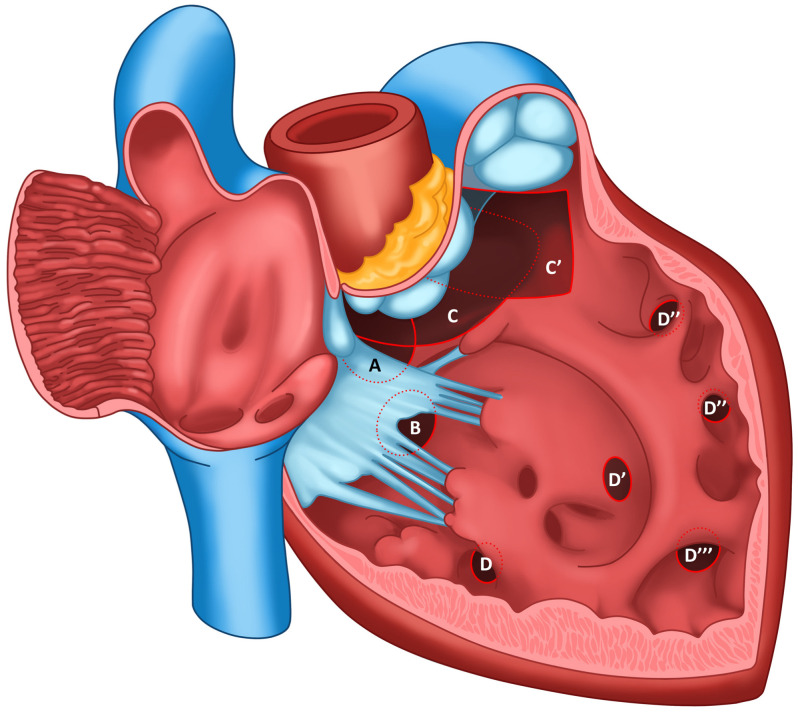
Cross-section of the heart as seen from the right atrium and right ventricle with the most important topographic structures. The cross-section of the right ventricle shows all the subtypes of defects according to The International Society for Nomenclature of Paediatric and Congenital Heart Disease Nomenclature with the most common phenotypes. A—central perimembranous ventricular septal defect, B—inlet ventricular septal defect with common atrioventricular junction, where the inferior rim of the defect has partially muscular borders, C—outlet ventricular septal defect between the limbs of the septal band (the dashed line indicates the border of the defect with this phenotype), C’—doubly committed, juxtaarterial outlet ventricular septal defect; defect located between the limbs of the septal band, but superior to the previous one, disrupting the fibrous continuity between the pulmonary artery and aortic valves. D, postero-inferior muscular defect; D’, midseptal muscular defect; D’’, apical muscular defect; D’’’, antero-superior muscular defect.

Despite extensive analysis, there were still some inconsistencies; therefore The International Society for Nomenclature of Paediatric and Congenital Heart Disease (ISNPCHD) established a new classification of VSDs that was accepted by the World Health Organization into the 11th iteration of the International Classification of Diseases. It focuses primarily on combining the previously mentioned strategies based not only on the exact location of the defect, but also on the structures bordering it, thus creating a common consensus. As a result, four main classes of VSD are listed: perimembranous central, inlet VSD with common AV junction, trabecular muscular, and outlet (Figure 3). In the terminology, each has subclasses that represent the phenotypes of the defect in question [4]. Central perimembranous defects are located below the posteroinferior limb of the septal band at the level of the anteroseptal commissure beneath the septal leaflet of the tricuspid valve on the right ventricular side. On the opposite side, they are located below the commissure between the right and noncoronary leaflet of the aortic valve. Inlet defects are located in the inflow portion of the RV below the anteroseptal commissure of the tricuspid valve, the posteroinferior limb of the septal band, and the medial papillary muscle. They may extend toward the membranous part of the interventricular septum and be associated with the overriding and straddling of the tricuspid valve and the malalignment of the atrial septum relative to the ventricle septum. Trabecular muscular defects are located in the apical part of the ventricle septum and have completely muscular borders. They are divided into midseptal, apical, postero-inferior and antero-superior muscular VSDs. Cases of multiple muscular VSD of the “Swiss cheese septum” type are also noted. The last type mentioned, outlet defects, represents the most complicated type of division due to the large number of possible phenotypes of occurrence concerning location and adjacent structures, as well as conduction pathways. They most commonly open into the RVOT between the anterosuperior and posterosuperior limb of the septal band. They can be associated with malalignment between the outflow septum and the apical part of the interventricular septum, by which further subclasses can be distinguished as outlet perimembranous defect, outlet muscular defect and doubly committed juxtaarterial defects with a muscular or fibrous posteroinferior rim [4]. However, a detailed explanation and description of each defect location with its visualization can be found in an announcement issued by ISNPCHD [4]. This subdivision allows a strategic approach to choosing the best possible surgical access and correction of the defect while minimizing the risk associated with severe arrhythmias [72]. We believe that standardizing the classification of VSD in veterinary cardiology will greatly improve communication between veterinary cardiology internists, but also contribute to an easier understanding of the location of VSD, and to determining the best strategy for patient management. Therefore, we fully recommend the incorporation of the classification established by ISNPCHD into veterinary medicine.

## 4. VSD in Dogs and Cats

**Figure 4:**
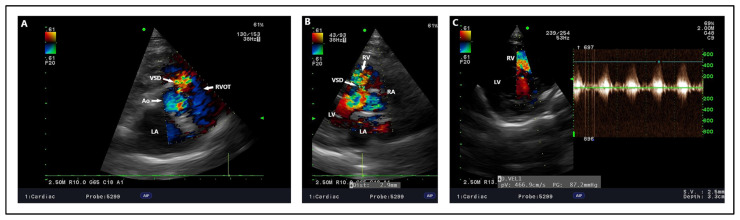
Outflow ventricular septal defect—dog, French Bulldog, four years. (**A**) Color Doppler, defect in the outflow portion as seen in the right parasternal short right axis view. (**B**) Color Doppler, defect with an estimated size of 2.9 mm in right parasternal four-chamber view. (**C**) Spectral Doppler, maximum velocity peak of ventricular septal defect seen from a modified left apical four-chamber view. Ao: aorta. LA: left atrium. LV: left ventricle. RA: right atrium. RV: right ventricle. RVOT: right ventricular outflow tract. VSD: ventricular septal defect.

**Figure 5:**
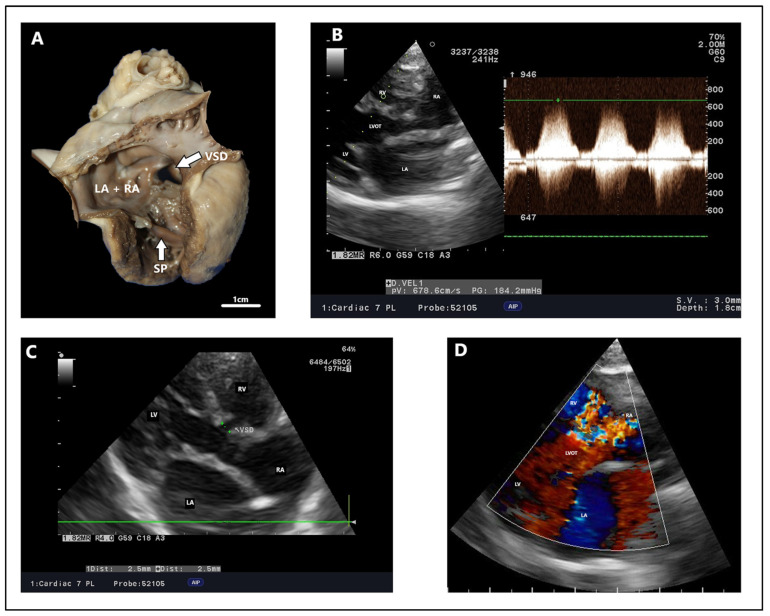
Atrioventricular septal defect with ventricular communication (inflow ventricular septal defect), domestic short-hair cat, four months. (**A**) Necropsy at six months due to congestive heart failure, with inflow ventricular septal defect visible on the dissected heart. (**B**) Spectral Doppler, maximum velocity peak of ventricular septal defect, modified right parasternal five-chamber view to estimate the maximum velocity peak. (**C**) Modified right parasternal five-chamber view to obtain the best estimation of the diameter of the defect. (**D**)—Color Doppler, characteristic “butterfly pattern” of atrioventricular septal defect with ventricular communication in the right four-chamber longitudinal axis. Multicolor in the upper part of the image indicates turbulent, rapid blood flow. LA: left atrium. LV: left ventricle. RA: right atrium. RVOT: right ventricular outflow tract. RV: right ventricle. SP: septal band. VSD: ventricular septal defect.

VSD in dogs is a relatively rare defect and represents between 4.8 and 14.4% of all CHDs in dogs [7,80,81,82,83,84,85]. It is placed fourth in terms of prevalence right after PS, subaortic stenosis (SAS), and patent ductus arteriosus (PDA) [7,84]. It can occur as a single defect (isolated VSD) or as a defect that is part of a multifactorial CHD. It is indicated that up to 50% of VSDs can occur with another heart defect [7,8]. Among the most common combined defects are VSD + PS [7], VSD + SAS, and VSD + double-chambered right ventricle (DCRV) [84], along with more complicated defects like VSD + DCRV + SAS + Tricuspid valve dysplasia [86] and Tetralogy of Fallot, which in addition to VSD consists of right ventricular hypertrophy, PS and malpositioned (overriding) aorta [7,8,84,87]. Because of the possibility of their simultaneous occurrence, it is important to perform a complete echocardiogram, even if the presumptive diagnosis is confirmed early in the examination. In cats, on the other hand, VSD is by far the most common CHD, comprising 20% to as many as 56% of all VSD cases [6,85,88]. This defect in cats is observed rather disparately, less frequently than other CHDs such as DCRV, dextroposition of the aorta, pulmonary atresia [88], as well as Tetralogy of Fallot, which is noted as amongst the most common when it comes to complex defects in cats [8]. The ventricular septal defect can also occur with atypical CHDs in cats, like double-inlet left ventricle with right ventricular hypoplasia [89] or tricuspid valve atresia [90]. No direct predisposition of breed among dogs and cats to this disease entity is indicated; however, a greater tendency to occur in French bulldogs, terriers [8,91], Border Collies [84], float-coated retrievers [80], and mongrel dogs [81] has been noted. As for cats, on the other hand, Maine Coone, Norwegian Forest Cat, and Sphynx have been mentioned most often, but the vast majority are domestic shorthairs [8,92]. In contrast, no predisposition of either gender to VSD is indicated in most of the studies. However, Schrope et al., 2015 [85], indicated a greater predisposition to VSD in feline males. Age of first diagnosis varies significantly and ranges from 1 to 157 months [7,81]. These data indicate that VSD is still underdiagnosed, and strengthen the concept of how important it is to routinely auscultate the patient during regular veterinary visits. It should be remembered that the earlier the defect is diagnosed, the higher the chances of prolonging and increasing the quality of life, due to the possibility of monitoring the animal later and implementing possible treatment. In both dogs and cats, perimembranous VSD is the most common as regards location, and this occurred in 71% of cases in dogs (Figure 4) and 79% in cats [8], which is consistent with the findings of other studies [85]. Second were VSDs located in the outflow tract, classified by other nomenclature as supracristal VSD [8]. In cats, there is also an indication of a greater predisposition to inflow pathway VSDs, which can also be classified as a partial AVSD with ventricle communication [85,93]. The difference between a classic VSD and a partial AVSD (Figure 5) is that there is a common AV valve annulus, but with two separate orifices from the atria to the ventricles, with a concomitant inlet VSD [65,94]. However, in dogs, this type, along with muscular VSD, is quite rarely described in veterinary medicine, being limited to single cases [9,12,95]. Another is Gerbode-type defects, where there is a perforation at the level of the AV or interventricular portion of the membranous part of the septum. In the case of the former, there is a direct connection between the LVOT, just below the aortic valve, and the right atrium, which can occur due to infective endocarditis and lead to an acquired VSD [96]. However, it should be kept in mind that infective endocarditis is not a common occurrence among affected dogs with VSD [67]. Cases of acquired Gerbode defect after traumatic events have also been described. Depending on the severity of the injury, we may note the spontaneous closure of the defect [97] or a worsening of symptoms due to the extent of the injury and the noted arrhythmic events, as well as secondary infective endocarditis [98]. In the case of a defect at the interventricular portion, this is rather said to be a congenital defect that involves a malformation of the tricuspid valve apparatus, including the frequent falling of the septal leaflet into the lumen of the defect and the formation of an intermediate connection between the LV and the right atrium [66,99]. In veterinary medicine, there are also reported cases of VSD in other animals, including rabbits [100], ferrets [101], goat [102], cattle [103], and horses, in which VSD, like in cats, is the most commonly reported CHD [104].

### 4.1. Clinical Findings

**Figure 6:**
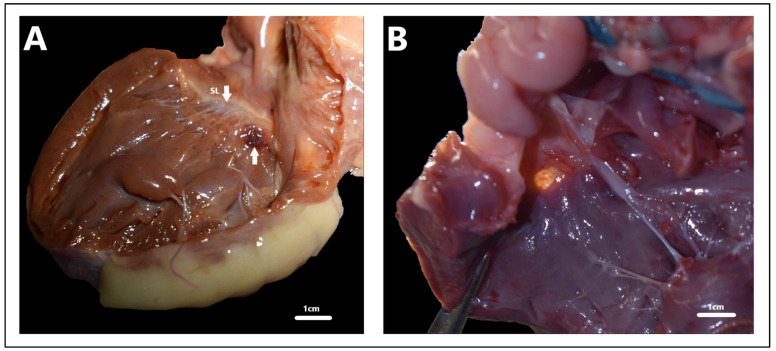
Central perimembranous VSD. Dog, mix-breed, fifteen years. (**A**) Right ventricular view, ventricular septal defect completely covered by the septal leaflet of the tricuspid valve. Adherence of the septal leaflet of the tricuspid valve to the VSD after infective endocarditis. White arrow—structural change due to vegetative forms of bacteria and myocardial lesion after infective endocarditis. (**B**) Same individual, view from the left ventricle—visible defect covered by the tricuspid septal leaflet from the right ventricle—asterisk. SL: septal leaflet.

Routinely performed auscultation of the heart during the first vaccinations allows the detection of a loud holosystolic, pansystolic, or systolic murmur located most often on the right side of the heart in cases of VSD [8,13,105]. The range of murmurs varies from III to VI/VI, but regardless of grade, they are mostly audible in all cases [8]. However, in cases of significant defects and associated right ventricular remodeling, the pressure between the ventricles may equalize, in which case the cardiac murmur is barely audible or disappears completely [8]. During the first visit, dogs are most often asymptomatic, thus not arousing the suspicion of the attending physician. Moreover, the absence of clinical signs may remain for the rest of their lives due to non-significant changes in cardiac hemodynamics in some animals with VSD [8]. In addition, there are sometimes cases in which the spontaneous resolution of isolated VSD occurs in both dogs and cats [106,107,108]. Such cases have also occurred after the surgical closure of a coexisting PDA [109]. Progressing over time, the defect can lead to the remodeling of the heart and pulmonary vessels, resulting in the appearance of clinical symptoms. These are mainly associated with respiratory symptoms, among which we can distinguish shortness of breath [12], dyspnea or tachypnea of varying severity [14,19,20,21], cough [21], thoracoabdominal asynchrony, [110], and exercise intolerance [8]. In bidirectional or reverse right-to-left shunts, additional symptoms related to cyanosis, seizures, collapse or ascites related to right-sided heart failure may develop. In addition, polycythemia and hyperviscosity can occur [111]. In addition, clinical symptoms can be exacerbated with concurrent infective endocarditis (Figure 6), thus resembling a septic condition [112]. We can then distinguish lethargy, reluctance to rise [66], fever, anorexia, depression [96], inappetence, dehydration [113], or even syncope [114]. It should be noted that VSD is in most cases the result of infective endocarditis, rather than its cause [66].

### 4.2. Echocardiography, Radiography, ECG Findings

Transthoracic echocardiography is considered the most optimal and non-invasive diagnostic method for VSD, helping to determine the location of the defect, its diameter, its maximum flow velocity, its hemodynamic significance, as well as the subsequent consequences of the defect resulting in morphological changes in the heart and pulmonary circulation. It is indicated as the gold standard in diagnosing congenital heart defects, having the highest specificity and sensitivity.

**Figure 7:**
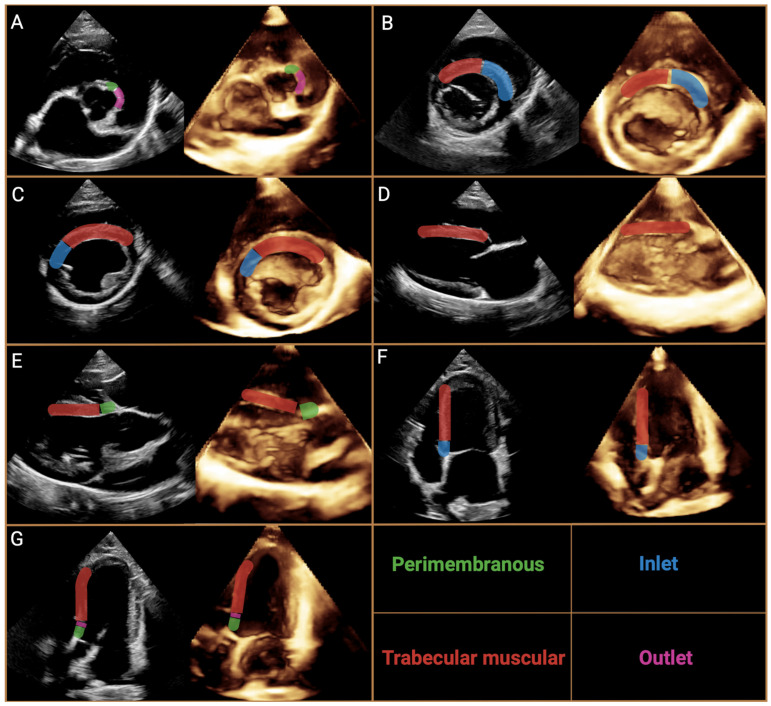
Proposed echocardiographic projections with explanations of the geography of the ventricular septal defect. (**A**) Right parasternal short-axis view at the level of the aortic valve—the optimal projection for obtaining a view of the outlet and perimembranous ventricular septal defect. (**B**) Right parasternal short-axis view at the level of the mitral valve—optimal for imaging trabecular muscular and inlet ventricular septal defect. (**C**) Right parasternal short-axis view at the level of the papillary muscles—a non-angled oblique projection, excluding the medial papillary muscle to optimize imaging on trabecular muscular and inlet ventricular septal defects. (**D**) Right parasternal four-chamber long-axis view—optimal for trabecular muscular ventricular septal defect. (**E**) Right parasternal modified five-chamber long axis view—optimal for predominantly central perimembranous ventricular septal defect. (**F**) Left apical four-chamber view—optimal for inlet and trabecular muscular ventricular septal defect. (**G**) Left apical three-chamber view—optimal for obtaining trabecular muscular and perimembranous ventricular septal defect. With a lot of experience of the examiner and maneuvering of the ultrasound transducer, it is also possible to capture the outlet ventricular septal defect. Green color—perimembranous ventricular septal defect. Blue color—inlet type ventricular septal defect. Red color—trabecular muscular ventricular septal defect. Purple color—outlet type ventricular septal defect.

**Figure 8:**
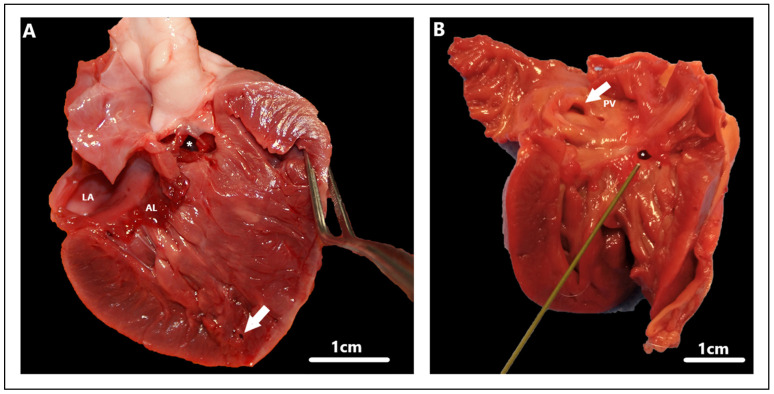
Outflow ventricular septal defect. (**A**) Dog, mixed breed, five months old. According to the consensus posted by The International Society for Nomenclature of Paediatric and Congenital Heart Disease nomenclature, this defect can be classified as a doubly committed juxta-arterial ventricular septal defect without malalignment with a muscular postero-inferior rim of the Outlet ventricular septal defect type. (**B**) Cat, Ragdoll, five months. Outlet muscular ventricular septal defect without malalignment. According to historical nomenclature, it may have been classified as a supracristal ventricular septal defect type. AL: anterior leaflet of the mitral valve. LA: left atrium. PV: pulmonic valve. Asterisk: ventricular septal defect located on the outflow tract of the left ventricle (outflow ventricular septal defect). White arrow: indicates a small, non-significant defect in the antero-superior muscular part according to The International Society for Nomenclature of Paediatric and Congenital Heart Disease nomenclature.

**Figure 9:**
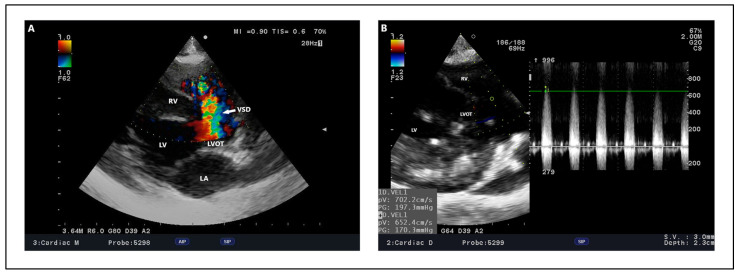
Central perimembranous ventricular septal defect, dog, Maltese, six years. (**A**) Color Doppler, defect in the membranous part of the interventricular septum located under the septal leaflet of the tricuspid valve seen from a modified right parasternal five-chamber view. (**B**) Spectral Doppler, maximum velocity peak of ventricular septal defect—7.02 m/s in modified right parasternal five-chamber view. LA: left atrium. LV: left ventricle. LVOT: left ventricular outflow tract. RV: right ventricle. VSD: ventricular septal defect.

In the case of VSD, the basis of echocardiography is to confirm the presumptive diagnosis, and further determine the exact location of the ventricular septal defect (Figure 7). After the morphological evaluation of the heart and determination of the location, it is important to determine the diameter of the defect, the maximum peak of shunt velocity, and the direction of blood flow through the defect. This determines future management, prognosis, and treatment options. VSD diameter can vary, but is usually in the range of 1.4–12 (mean 3.9 mm) for dogs and 1–12 mm (mean 3.5 mm) for cats [8,115]. Despite careful measurements, overlooking an additional VSD is possible, and its discovery occurs only during surgery to close a large defect [19] or at post-mortem examination (Figure 8). In addition, there is a risk of underestimation during transthoracic echocardiography measurements, which can be corrected preoperatively with transesophageal echocardiography, and the VSD diameter itself can vary up to 2 mm [13]. Depending on the size of the defects and the pressure difference between the left and right ventricles, different maximum velocities through the shunt are distinguished. These can range from 3 to 6.75 m/s in dogs and 0.57 to 6.72 m/s in cats, but most animals show flows in the range of 4.8 to 6.75 m/s [8] (Figure 9), which has been confirmed by other investigators [13,20,21,115]. Due to the large difference in pressure gradient between the chambers, left-to-right flow is usually distinguished. However, it varies depending on the severity of the defect and the morphological changes already present in the heart, resulting in the adaptation of muscle tissue to the body’s requirements. Therefore, the pressure differential can be variable, and range from 43.6 to 157 mmHg [13,18,19,115]. It is worth noting that other cardiac defects can significantly affect both the intensity of velocity and the pressure gradient between ventricles such as PS or PDA. Such conditions lead to RV pressure overload [19,116], resulting in a decrease in these values, which, in combination with cardiac auscultation, can result in a softer heart murmur [117]. On the other hand, defects such as SAS can lead to increased LV pressure, thereby raising the pressure gradient between LV and RV [118]. Therefore, it is important to be mindful that when confirming one CHD, a full echocardiogram should be performed, even after the main diagnosis has been made. Further, with a large and long-standing defect, flow reversal and bidirectional or right-to-left shunt can occur [12,66,119]. This results in the remodeling of the pulmonary arteries, causing hyperproliferation and obstructive changes of endothelial cells, smooth muscle, and fibroblasts. Pulmonary vascular resistance is significantly altered, and the pulmonary circulation begins to overtake the peripheral circulation, clinically manifesting as cyanosis [120]. These changes are referred to as Eisenmenger’s syndrome [110], which is most common in humans as a consequence of VSD [121]. In addition to other cardiac defects, the chronicity of VSD alone can lead to right ventricular (RV) pressure overload and the development of Eisenmenger syndrome. For large, non-restrictive untreated defects in humans, this process develops around the first year of life [122], while in dogs and cats it develops much faster; as early as 6 months of age, Eisenmenger syndrome can be completely developed [123]. This process, extended over time, results in cardiac remodeling associated with RV myocardial hypertrophy, RA enlargement, pulmonic artery dilation without any stenotic lesion, and the flattening of the interventricular septum and paradoxical septal motion during systole and diastole, suggesting right ventricular volume and pressure overload with pulmonary hypertension [124,125]. Initially, due to the high-pressure left ventricle, during systole, blood is pumped through the defect into the low-pressure right ventricle, which, over time, results in changes associated with its remodeling. During the development of Eisenmenger syndrome, the pattern of blood flow changes into bidirectional. During the early systolic phase, blood from the left ventricle is pumped through the defect into the right ventricle, weakening and equalizing during the late systolic phase. In contrast, during early diastole, blood pressure in the left ventricle drops suddenly, driving blood from the right ventricle into the opposite side of the interventricular septum. When the late diastolic phase occurs, the pressures in both ventricles equalize, resulting in the relative cessation of blood flow through the defect. Subsequently, the resistance in the pulmonary vascular bed is high enough that the pressure in the right ventricle during systole begins to exceed that in the left ventricle, resulting in a complete reversal of flow to a right-to-left-shunt pattern [126,127]. In addition, the blood flow patterns are equivalent to changes in the pressure gradient between the ventricles, with the early stage of the disease having a high-pressure flow, resulting in a higher pressure gradient. With progressive right ventricular changes, secondary to the developing Eisenmenger syndrome, the gradient begins to decrease significantly during systole, indicating disease progression [128]. Therefore, to calculate the severity of the defect, several measurements should be evaluated. In addition to the previously mentioned maximum peak velocity through the shunt and the pressure gradient between ventricles, an interesting determinant indicating the hemodynamic relevance of blood flow through the shunt and thus the contribution of the pulmonary circulation to the peripheral circulation is the Qp:Qs ratio. In humans, this ratio is one of the decisive indicators of a possible surgical procedure that can be performed to close the defect. It is accepted that a Qp:Qs ratio greater than 1.5 and left heart volume overload may be indications for surgery [129,130]. It is also believed that this ratio is repeatable and reproducible in dogs, and its average value in healthy individuals, as in humans, equals 1 [131]. In dogs and cats with VSD, a Qp:Qs ratio < 1.5 indicates a mild restrictive VSD, between 1.5 and 2.5 is moderately restrictive, and above 2.5 suggests a severe, non-restrictive VSD [8]. A correlation was indicated in dogs, where a higher Qp:Qs ratio was significantly correlated with the onset of clinical signs, which was not confirmed in cats [8]. In addition, the ratio was found to be useful in assessing circulatory function after the procedure, where it returned and remained close to reference values, indicating the success of the procedure itself, as well as the recovery period [15,16,21]. With large defects, the Qp:Qs may prove useful when monitoring disease progression because it could help track the progression of right-sided pressure overload and pulmonary hypertension, which may lead to a gradual decrease in Qp:Qs. This would allow for a better evaluation of the hemodynamic consequences of long-standing VSD. However, it should be remembered that this is not always feasible, especially for defects such as PS or SAS [14]. Another indicator used is the VSD:Ao ratio. As with Qp:Qs, a VSD/Ao ratio greater than 0.4 is associated with a higher percentage of clinically affected dogs, but this has not been confirmed in cats [8]. However, this is due to the small number of clinically affected cats in this study, so further studies verifying both Qp:Qs and VSD:Ao ratio should be conducted among these animals. Additional measurements such as evidence of left heart volume overload may be crucial when it comes to deciding on surgery [8,19]. In lesions associated with the left side of the heart, the enlargement of the LV and left atrium can occur [10,15,18], as well as mitral regurgitation [16,18]. In defects located close to the AV junction, just beneath the sinus of Valsalva, the structures supporting the aortic ring are weakened, contributing to aortic regurgitation. This process, when extended over time, causes hyperplasia and rolling of the cusps of the aortic valve, exacerbating regurgitation, which may predispose the individual to infective endocarditis [17,113,132]. To assess the degree of pulmonary hypertension, the gold standard is to catheterize the pulmonary artery and assess the pulmonary arterial pressure. Nevertheless, this procedure is associated with high invasiveness, so it is not routinely performed in veterinary medicine. An indirect method of assessing pulmonary hypertension is the assessment of tricuspid regurgitation, after which the maximum velocity peak of tricuspid regurgitation is substituted into a modified formula of Bernoulli’s equation, which gives us a rough estimate of pulmonary artery pressure [133]. However, this is not the best method when using pulmonary hypertension assessment. Therefore, measurements based on Qp:Qs values, the pressure gradient between the ventricles or VSD/Ao ratio together with echocardiographic changes in the heart allow for an accurate assessment of disease progression when the individual is monitored regularly.

A possible option when undertaking a VSD assessment of the pulmonary vasculature and cardiac structure is using radiographs. Using these two imaging techniques, the extent and significance of the lesions can be determined even more precisely, and the impact of the defect on the pulmonary circulation can be assessed. Assessment is based primarily on an evaluation of heart size using the vertebral heart scale (VHS). With small VSDs, the overall silhouette as well as the size of the heart may remain unchanged, within reference limits [15]. On the other hand, large defects may lead to significant changes in heart size, where the VHS may oscillate between 11.25 and 14.5 depending on the severity of the defect, time to first diagnosis, and the treatment implementation [11,14,18]. The abnormalities that can be seen in the radiograph in animals with significant lesions are partial or general cardiomegaly, pulmonary overperfusion, pulmonary venous congestion, and loss of the cranial waist of the cardiac silhouette [10,11,12,13,18,19,21]. However, X-rays not only determine the characteristics of the lesions, but can also serve as a tool to control the progression of the changes and the recovery period after the surgical closure of the defect [11]. Nevertheless, this approach has low sensitivity, so it is considered a possible additional diagnostic tool. Variations such as acquired heart disease, comorbid CHD, or even racial differences in thoracic and myocardial structure [134] can affect the subjective assessment of the examiner.

At the time of the decision for surgery, ECG recordings are advisable. During anesthesia application, the respiratory–circulatory center is depressed, which predisposes the induvial to life-threatening arrhythmias. In addition, due to the significant enlargement of the heart cavities, the risk of anesthesia becomes significantly higher, so continuous anesthetic care is a requirement for this type of surgery. In most cases, ECG recordings of patients referred for surgery do not show significant deviations from physiological sinus rhythm. Still, there may be isolated cases in the form of occasional isolated ventricular premature contraction [13]. Due to enlargement of the cardiac chambers, electrical left axis deviation, and high R wave in the case of LV enlargement/hypertrophy and electrical right axis deviation and deep S wave in the case of RV enlargement/hypertrophy may occur [69]. In cases of reverse right-to-left shunt, changes including pacemaker wandering, inverted QRS complex on second lead, and electrical right axis deviation suggestive of RV hypertrophy can be observed [110]. With severe VSD complications and the onset of infective endocarditis, AV conduction abnormalities occur, resulting in a third-degree AV blockage that is non-responsive to atropine [66,114]. Regarding the previously mentioned partial AVSD with ventricular communication, most cases resulted in an intraventricular conduction disturbance in the form of right bundle branch block (RBBB) or partial RBBB and, less frequently, left anterior fascicular block. This is thought to be related to malposition and delayed impulse propagation in the intraventricular conduction pathways due to septal malformations and the close contact of the defect with the AV node [93]. ECG has low specificity and sensitivity, so it can serve as an additional tool in assessing the extent of myocardial lesions and prognostic evaluation. Nevertheless, it should always be performed during an echocardiogram to determine any congenital defect.

### 4.3. Management of VSD

In most cases, the diagnosis of VSD is completely coincidental, usually during routine vaccination and heart auscultation. Such dogs or cats are usually asymptomatic and referred for echocardiography. The second possibility for determining the diagnosis of the defect is the owners’ concern about the onset of clinical signs. It has been indicated that the average age of onset of clinical signs is about 12 years [8]. Still, it should be remembered that this depends primarily on the severity of the defect. In the case of the former, treatment is usually not implemented, and the owner is advised to observe the animal and perform more frequent cardiac monitoring to keep track of the changes. In the case of significant cardiac lesions or the onset of clinical signs, treatment based on conventional methods is recommended, in the first instance. Angiotensin-converting enzyme inhibitors and diuretics are often prescribed. Still, it must be remembered that the selection of appropriate drugs is based on clinical signs and symptoms, as well as additional tests. However, these cases are not common, and these animals do not take medications for most of their lives [8]. In the case of large, non-restrictive defects and no decision on surgical treatment, there is a high risk of developing Eisenmenger’s syndrome, which is equivalent to pulmonary hypertension and secondary cardiac lesions. The pharmacological treatment of choice is the introduction of phosphodiesterase type V inhibitors (sildenafil), myelosuppressive drugs (hydroxyurea), and interventional phlebotomy when the hematocrit level increases significantly. The careful monitoring of such patients, the modulation of drug dosage and a rapid response to worsening clinical symptoms can, with great success, keep such patients clinically stable for a long period of time [125,135].

Animals diagnosed based on clinical signs are usually of advanced age, which typically eliminates the patient from the possibility of a surgical procedure. In contrast, in young patients diagnosed with a VSD, or with clinical signs due to significant morphological changes in the heart and pulmonary circulation, the surgical closure of the VSD is a possible treatment option. The decision for surgery should be taken with special consideration of several guidelines considering echocardiography, X-ray examination, and the age and clinical condition of the patient. Although data on surgical VSD closure in companion animals are limited, there are reports of successful surgery with long-term patient outcomes. Surgical techniques have been mainly derived from human medicine, but not all of them are feasible in dogs and cats, often due to the much smaller sizes of these animals compared to humans. There are several options for VSD closure, ranging from more invasive methods and possible major complications to less invasive methods that require appropriate training and special equipment. On the other hand, each is associated with certain limitations, so when considering the best surgical technique, the patient’s records should be carefully examined, and a possible option selected based on these [136]. Considering the rapidly growing branch of cardiology and cardiac surgery in veterinary medicine, we believe that surgical options for treating VSD will be more widely used. In addition, the increased awareness of CHD in dogs and cats is giving rise to more and more screening, and thus the faster detection and more comprehensive evaluation of CHD.

#### 4.3.1. Pulmonary Artery Banding

This method is a palliative treatment option, where the defect is not closed, but the negative hemodynamic effects generated by the left-to-right shunt through the VSD are counteracted. It can be described as a method that pathologically equalizes the pressure difference between the chambers, slowing the nature of morphological changes in the heart [136]. The main risk of this technique is the possibility of over-clamping the pulmonary trunk and consequently reducing flow via the shunt. It is said that it is optimal to clamp the artery by approximately two-thirds of the vessel diameter, but in the case of puppies or kittens, this should be done with a high level of caution. In such cases, incomplete clamping or the use of a dilatable artery band should be considered [19]. In some cases, simply inserting the ligature without clamping may have the desired effect after maturity [19]. Therefore, it is important to optimize the timing of discontinuation of compression to increase the chances of long-term success. Therefore, there are four critical moments indicating correct clamping: (1) decreasing pulmonary arterial pressure toward 30 mmHg posterior to the site of clamping, which will slow down pulmonary vascular remodeling and the onset of Eisenmenger syndrome; (2) increasing systolic arterial pressure to reach the plateau phase, the aim of which is to achieve an increase of 10–20 mmHg without causing bradycardia or a drop in arterial blood saturation below 90%; (3) decreasing pulmonary artery blood saturation by about 5–10%; (4) decreasing max velocity peak shunt towards but not below 2.5 m/s [19,136,137]. Although the procedure is associated with considerable invasiveness, it appears to be a reasonable method when used as a palliative treatment option, as previously clinically affected cats become asymptomatic up to 40 months after surgery [19]. In addition, this method may be applicable in cases of concomitant PDA, due to the possibility of closing the Botall’s duct as well as placing an arterial trunk band during the same procedure, without performing additional surgical accesses [19]. In addition, attempts to close the defect by using various occluders may be ineffective in cats with a large defect due to possible obstruction of the LVOT caused by the disc protruding into the left ventricular lumen [20]. Due to the small size of the feline heart, VSD closure seems difficult to achieve; therefore, when surgery is required, pulmonary artery banding seems a reasonable option. However, it should be noted that the cardiac changes continue to progress, but will be significantly slowed, providing the opportunity to live longer in greater comfort.

#### 4.3.2. Open Heart Surgery

These procedures involve the greatest invasiveness of all VSD repair methods. This is due to the high level of soft tissue interference during the performing of thoracotomy, and the incision of the pericardial sac and the heart. A cardiopulmonary bypass is used to maintain vital functions, and to reduce bleeding and cardiac motions, allowing for a more precise approach. One interesting, albeit controversial, alternative is cross-circulation cardiopulmonary bypass, whereby the arterial blood of another donor dog is continuously transfused into the operated animal. The risks are primarily associated with the possibility of hypoxia in some parts of the brain, with consequent death shortly after surgery [138]. Other, less severe risks may include hypokalaemia or peripheral edema [10]. Currently, the use of cardiopulmonary bypass with cross-circulation is avoided for safety reasons. The two most common and widely used accesses are right atrial incision and right ventricular incision. The former is mainly used to close defects located under the septal leaflet of the tricuspid valve, and therefore in the membranous part of the septum [136]. Access through the right ventricular incision can also be performed for the closure of perimembranous VSDs; however, due to the larger surgical field and the possibility of accessing other locations, it is also considered for the repair of outflow VSDs [136]. However, due to the significantly longer hospitalization and intensive care time and the greater oxygen requirements in the RVI group, the authors indicate that right ventricular incision is minimally more invasive than right atrial incision, and therefore the closure of perimembranous VSD should be preferred via right atrial incision [105]. VSD closure itself is performed by applying and suturing a special patch tailored to the size of the defect. The critical point of VSD closure is the possibility of generating arrhythmias in the form of RBBB, premature ventricle contraction, or second-degree AV block, which should be resolved a few days after the procedure [105]. Persistent arrhythmia may also occur as a result of damage to intraventricular conduction pathways during defect repair [105]. These procedures can be combined to repair other significant defects or morphological changes, such as myocardial tumors eligible for removal or aortic valve myxoma [139]. However, it should be noted that this type of heart surgery involves a high degree of invasiveness in the myocardial tissue, so it may be associated with death during surgery or as a result of postoperative complications [140]. An alternative option to open heart surgery is the introduction of a hybrid technique, also involving access to the heart via thoracotomy, but VSD closure itself is performed with dedicated occluders, as are used in the percutaneous transcatheter closure of the defect. These devices, in the context of humans, are designed for patients weighing at least 8 kg [141], but it is possible to perform this procedure in patients weighing less than 5 kg using this technique [142]. In the case of veterinary patients, such a solution seems to have particular validity, especially as these patients are often small in size. Furthermore, there are reports of the success of the purported procedure and the long-term positive effects of the surgery in dogs [18].

#### 4.3.3. Percutaneous Transcatheter VSD Closure

The least invasive procedure among those mentioned is percutaneous transcatheter closure of the VSD. The method itself involves inserting a catheter usually through the femoral artery, and closing the defect on the beating heart. Despite its low invasiveness, this method is not commonly used, but this is most likely due to the requirement for specialized equipment and the experience of the operator surgeon and the entire team. Despite this, there are already several reports in the veterinary medicinal literature describing cases of defect closure using various techniques, including percutaneous closure, with long-term follow-up (Table 2). In addition to the previously mentioned weight requirements, another absolute contraindication is the closure of the defect less than 3 mm below the aortic valve. VSD-implanted occluders located below this value can indeed interfere with the aortic valve apparatus, leading to severe AR [143]. Even more so, devices implanted within 3 mm can already cause mild, but not hemodynamically significant, AR [15,17]. At present, there are few reports of percutaneous VSD closure in veterinary medicine. This makes the use of different occluders more interesting, and the choice of each occluder is generally tailored based on VSD location, patient size, and device characteristics. One of these is the Amplatzer duct occluders dedicated specifically to PDA closure, but these are also an option for VSD treatment. One limitation of their use is their relatively limited size range, so for defects above 5.5 mm, there can be considerable difficulty in locating the right device [144]. In addition, it should be considered that the discs of the device should not affect the mechanics of the aortic and tricuspid valves, causing their iatrogenic regurgitation. Although this technique is considered minimally invasive, it also carries the risk of certain complications. One of these is the possibility of the incomplete closure of the defect, resulting in residual shunt flow [17]. Another complication is the possibility that the device may dislodge from the defect and fall into the ventricular lumen, generating a recurrent VSD. This is not a common finding; however, it is possible for it to occur when an inadequately sized occluder is used, or when an attempt is made to close another coexisting CHD, such as PDA, leading to a rapid change in pressure and the possibility of device dislodgement from the defect [14]. In addition, with reference to a meta-analysis of the use of this technique in humans, it can be said that the procedure itself has a high success rate, with a low number of postoperative complications. However, it should be remembered that complications like arrhythmic events, including bifascicular block, incomplete RBBB, complete RBBB, and first-degree heart block can occur as transient or permanent events [145]. Severe arrhythmias such as complete atrioventricular block can also occur shortly after surgery, and in the long-term follow up [146]. The choice of occluder should be made based on the features of the individual case, but several types of devices have now been designed. One study comparing three types of occluders in perimembranous VSD repair indicated that the Amplatzer Duct Occluder II displayed the best properties in terms of softness, flexibility, and time of implantation [147]. In pursuit of providing the safest services possible, innovative solutions are being developed every year to reduce the risk of intra- and postoperative complications. For this reason, new devices have been developed based on modern solutions, such as the KONAR-MF occluder, which during clinical trials showed a low risk of postoperative complications, high flexibility, high comfortability with septal defect [148,149], and most importantly, sizes ranging between 4 and 7 Fr, which in the case of veterinary patients is of considerable importance. Nevertheless, patients need to be specifically selected for a particular type of occluder. Further, amplatzers made of biodegradable materials that rapidly endothelialize and absorb, reducing the possibility of dangerous arrhythmias, may represent a future solution, but research is still needed to determine their full safety [150,151]. In addition, their use against growing defects in very young individuals is such that, during maturation, the biodegradable material does not interfere with myocardial growth and function [152]. However, there are only a few clinical reports on their use, and their mechanical properties, optimal size for veterinary patients for percutaneous delivery, or potential autonomous deployment have not yet been developed for commercial use [153]. Therefore, further studies are needed into their application and permanent introduction into human or veterinary medicine. Additionally, it should be noted that the use of the percutaneous closure technique in cats has some limitations, mainly due to the small size of the heart and the possibility of obstruction of the ventricular outflow tracts [20]. Despite some risk, patients in whom the percutaneous closure of the VSD has been attempted have mostly shown the resolution of pre-existing clinical symptoms, as well as the regression of morphological changes in the heart (Table 2).

Patients who have successfully undergone VSD closure surgery require pharmacological postoperative care, so it is advisable to use specific management protocols. Depending on clinical symptoms and presentation at initial visits, the mode of management may vary slightly. Hospitalization from 24 to 48 h after the completed procedure is essential for monitoring the patient’s health [13,14]. The length of hospitalization primarily depends on possible postoperative complications. The usual complications are transient atrioventricular blocks or other forms of arrhythmia, which usually resolve spontaneously from a few hours to a few days [13,15]. Postoperative care is based primarily on the absolute restriction of movement for up to 4 weeks, and the performance of antibiotic therapy and anticoagulant treatment [18], due to the possibility of clot formation with occluder usage in dogs [154,155]. Treatment related to analgesic or antiarrhythmic effects should be selected in terms of individual patient needs. The majority of patients in whom percutaneous VSD closure is successful exhibit promising results at follow-up (Table 2). Therefore, this solution is likely to be a prospective option for patients with a large, non-restrictive VSD.

**Table 2:** Cases described in the literature of the surgical treatment of VSD in dogs and cats.

Author	Breed	Age (Months)	Clinical Signs	Ventricular Septal Defect Type and Diameter (Millimetres)	Maximum Ventricular Septal Defect Velocity Peak	Treatment Method	Echocardiographic Findings		Radiographic Findings	Electrocardiographic Findings	Clinical Outcomes
							Before Procedure	After Procedure			
[10]	Cavalier King Charles Spaniel	3	The loudest murmur on the right side	Perimembranous, 10	NM	Open heart surgery with cross-circulation	NM	After surgery within normal limits, no residual shunt flow	Left-sided cardiomegaly, pulmonary over-perfusion, prominent main pulmonary artery, two months after the procedure—normal	After surgery persistent RBBB	Improved exercise tolerance, soft systolic murmur at the level of the TV after 2 months
[11]	Shiba Inu	2	III/VI right-sided holosytolic murmur	Partial AVSD with ventricular communication, NM	NM	Open heart surgery under cardiopulmonary bypass	LV and LA enlargement, MR with cleft of septal leaflet, L-to-R shunt	Mild MR, no residual shunt, reduced LVIDd	VHS before procedure—13.7, VHS after procedure—11.3	Normal	Clinically normal after the year
[12]	Cavalier King Charles Spaniel	7	Shortness of breath when excited, V/VI right-sided systolic murmur	Muscular, 10	NM	Percutaneous transcatheter closure (Amplatzer Muscular VSD occluder)	RV enlargement with wall thickness, bidirectional shunt flow	No residual flow through the device, Trivial TI, Trivial MI	Right ventricle enlargement, Loss of the cranial waist of the cardiac silhouette	NM	Clinically normal after 2 years
[13]	English Sheepdog	12	IV/VI right-sided holosystolic murmur, reduced exercise capability	Perimembranous, 6	4.8 m/s	Percutaneous transcatheter closure (Amplatzer occluder)	Qp:Qs: 1.76, EDVI—215 mL/m^2^, L-to-R shunt, Pressure gradient 92.2 mmHg	No residual flow through the device	Mild cardiomegaly with over-circulation of pulmonary vasculature, pulmonary venous congestion	VPCs—isolated ventricular premature beats, ECG after treatment was normal	Clinically normal after 1 month
[14]	Irish setter	7	Mild tachypnea, respiratory effort after strenuous exercise, VI/VI left basilar systolic murmur	Muscular, 12	Bidirectional flow	Percutaneus transcatheter closure (amplatzer post-infarction muscular ventricular septal occluder)	RV hyperthrophy with systolic and diastolic septal flattening, moderate PI, severe post stenotic main and right pulmonary dilatation	Persistent mild L-to-R flow 5 months after procedure	Generalized cardiomegaly, VHS 11.25, dilation of the main pulmonary artery segment	NM	Died after 5 months after surgery due to acute neurological signs and respiratory failure
[15]	Beagles	1	III/VI right-sided systolic murmur	Perimembranous, NM	NM	Percutaneous transcatheter closure (detachable coil)	Qp:Qs—1.2, L-to-R shunt, aortic maximum velocity peak 0.93 m/s	Qp:Qs—1, aortic maximum velocity peak 1.20 m/s, small AR	No evidence of cardiomegaly, after the procedure, there was no evidence of abnormal cardiac silhouette	Normal rhythm	Diminished heart murmur
[16]	Mongrel	12	V/VI right-sided holosytolic murmur	Perimembranous, 7.8 from LV, 3.4–4.7 from RV	4.7 m/s	Percutaneous transcatheter closure (canine duct occluder)	LA/Ao 1.8, Qp:Qs—1.7, Mild MR, LVIDdN—1.87	LA/Ao—1.41, Qp:Qs—1.1, LVIDdN—1.69	NM	NM	Clinically normal after 2 years
[16]	Staffordshire terrier	6	VI/VI right-sided systolic murmur	Perimembranous, 9.69 from LV, 5.78 from RV	NM	Percutaneous transcatheter closure (canine duct occluder)	LA/Ao 1.7 LVIDdN—1.79 Mild MR, PR, AR,	Qp:Qs 1.2, LVIDdN 1.66	NM	NM	Clinically normal after 8 months
[18]	Bichon Fries	4	Tachypnoea during exercise, VI/VI right-sided systolic murmur	Outlet, 4.4	3.3 m/s	Percutaneous transcatheter closure (amplatzer duct occluder II)	LA/Ao—2.15, MR, Qp:Qs 2.8, Pressure gradient 43.6 mmHg LVIDd—26 mm LVIDs—15 mm	17 days after the procedure: LVIDd—24 mm LVIDs—15 mm	Pulmonary vessel enlargement, cardiogenic edema in the caudal lung lobe, VHS before procedure—12.2 VHS 17 d after procedure—10.3	NM	Clinically normal after 4 months
[19]	Domestic short hair cat	3	V/VI right parasternal systolic murmur	Perimembranous, 1.9	3.7 m/s	Pulmonary artery banding	LA/Ao 1.94, Qp:Qs 2.4, L-to-R, Pressure gradient 56 mmHg, LVIDd—15 mm, moderate concentrate thickening of RV	LA/Ao—2.1, VSD maximum velocity peak 2.6 m/s, Pressure gradient 26 mmHg, mild-to-moderate RV concentric hypertrophy	NM	NM	Clinically normal after 40 months
[19]	Domestic short hair cat	10	V/VI right parasternal systolic murmur	Perimembranous, 4	3.9 m/s	Pulmonary artery banding	LA/Ao 2.7, Qp;Qs 2.1, L-to-R flow, Pressure gradient 61 mmHg, LVIDd—26.2 mm	VSD maximum velocity peak 3.4 m/s, pressure gradient 37 mmHg, moderate dilation of LVIDd	Generalized cardiomegaly, severe LA enlargement, marked over-circulation of the pulmonary vessels	NM	Clinically normal after 9 months
[19]	Domestic short hair cat	1	Activity intolerance, resting tachypnea, V/VI right parasternal systolic murmur	Perimembranous, 2.5	2 m/s	Pulmonary artery banding	LA/Ao 1.9, LVIDd dilation, MR, PR, TR	LA/Ao 1.4, LVIDd—17.7 VSD maximum velocity peak 3.6 m/s, Pressure gradient 52 mmHg,	NM	NM	Clinically normal after 9 months
[20]	Maine Coon	13	Exercise intolerance, respiratory distress, abdominal and mouth breathing, tachypnea	Perimembranous, 7.6	4.9 m/s	Percutaneous transcatheter closure/pulmonary artery banding	Qp:Qs 3.82, LVIDd—34.4	Qp:Qs 2.28, volume overload, CHF after 8 months	NM	NM	Clinically normal after 8 months
[21]	Domestic short hair cat	8	VI/VI systolic right-sided murmur, severe tachypnea, dry cough	Perimembranous, NM	5.48 m/s	Pulmonary artery banding	Qp:Qs 3, MR, LVIDd 19 mm, LVIDs 12 mm	Qp:Qs 1.5 after 14 months	Left atrial enlargement, moderate RV enlargement, mild to moderate enlargement of the pulmonary vasculature, mild interstitial pattern in the cranial lung lobes, bronchointerstitial lung pattern in the perihilar region and caudodorsal lung fields	NM	Clinically normal after 14 months
[156]	Maltese and poodle mix	5	V/VI systolic right sided murmur, mild precordial thrill, jugular vein distension, nonproductive cough	Muscular, 5	1.1 m/s in systole, 0.9 m/s diastole	Transcatheter occlusion	Right ventricle enlargement with interventricular septal flattening, LVIDd/RVIDd < 1, MPA/AO ratio 1.41, RPA/AO ratio 0.72, PR 4.3 m/s (>74 mmHg), Qp:Qs—2.75	LVIDd/RVIDd ratio 2.1, Ao/MPA ratio 0.81 Ao/RPA ratio 0.6 PR 1.84 m/s (13.5 mmHg), Qp:Qs ratio 1.3	Enlargement of right cardiac silhouette (VHS 11.6), dilation of the main pulmonary artery and pulmonary vessels CVC/VL 1.8	Sinus tachycardia (180–200 bpm), Deep S-wave	Clinically normal 9 months after procedure

Ao: aorta. AR: aortic regurgitation. AVSD: atrioventricular septal defect. CVC/VL: caudal vena cava to vertebral length ratio. ECG: electrocardiography. EDVI: end diastolic volume index..L-to-R: left-to-right shunt. LA: left atrium. LA/Ao: left atrium to aorta ratio. LV: left ventricle. LVIDd: left ventricular internal end-diastolic diameter. LVIDdN: normalized left ventricular internal diameter in diastole. LVIDs: left ventricular internal end-systolic diameter. MPA: main pulmonary artery. MR: mitral regurgitation. NM: not measured. PR: pulmonic regurgitation. Qp:Qs: pulmonary (Qp) to systemic flow (Qs) ratio. RBBB: right bundle branch block. RPA: right pulmonary artery. RV: right ventricle. RVIDd: right ventricular internal end-diastolic diameter. TR: tricuspid regurgitation. VHS: vertebral heart score. VSD: ventricular septal defect.

## 5. Long Term Follow-Up

A small, restrictive VSD is associated with a relatively good prognosis, not requiring pharmacological treatment for a long time. In the most comprehensive study regarding VSD in dogs and cats, it was estimated that in cats, the median clinical manifestation was 12 years. In dogs, due to the lack of clinical symptoms becoming apparent in most animals, a median was not established [8]. The median age at death was approximately 12 years for both groups; however, only three individuals (of the eight with clinical signs) were associated with left-sided congestive heart failure, while only one was closely associated with VSD. These data remain consistent with the results of other studies [115]. The remaining five symptomatic animals required pharmacological treatment based on ACEI and diuretics [8]. This suggests that the quality of life of patients with VSD is satisfying, and the defect is well tolerated for most of life in affected individuals. The early onset of symptoms is primarily related to the size of the defect and its hemodynamic significance. Clinical signs before surgery, measurements of echo, X-ray, and ECG before and after surgery if available, and the evaluation of the condition at the last visit before publication of the case, are presented in Table 2. The data presented in the table suggest that in cases of successful interventions, patients remained clinically asymptomatic and hemodynamically stable at the time of publication of the papers. Therefore, it is of utmost importance to use an appropriate diagnostic approach, along with an optimal treatment strategy designed individually for each patient, if required.

## 6. Conclusions

VSD can be described as a remarkable defect, primarily due to its polyphenotypic nature, which often makes it difficult to predict in terms of subsequent prognosis. Another phenomenon of this defect is its coexistence with other CHD such as PS, PDA, or SAS, thus being a complex defect in about 50% of cases. It is also part of more complex disease entities such as the Tetralogy of Fallot. Most cases do not require surgical or even pharmacological intervention, with a high survival rate. Nevertheless, over time, the exacerbation of the disease can occur because the underlying nature of this defect is a progressive morphological alteration of the heart as well as the pulmonary vessels, leading to severe cardiopulmonary changes. During this phase of the disease, clinical symptoms become apparent, significantly affecting the quality and comfort of patients’ lives. Therefore, early diagnosis is crucial for patient monitoring and management. Due to the characteristic (usually right-sided) murmur, a presumption of VSD may be declared on auscultation during the first vaccinations. In this case, echocardiography is conclusive, but other examinations with radiography and ECG will provide additional information about the patient. In addition, making a diagnosis regarding the presence of a VSD should not end the cardiac examination at this stage, due to the possibility of coexisting congenital anomalies that may affect further decisions about the patient. Constantly developing treatment techniques allow the selection of appropriate treatment options, including surgical closure of the defect, which seems to be the best future approach for patients with an absolute indication for surgery. A quick and accurate diagnosis will determine further management and, in the most severe cases, accelerate the decision regarding possible surgery. For this reason, it is very important to make owners as well as internists aware of how crucial it is to examine patients thoroughly during their first visits. The monitoring of patients with a diagnosed VSD should take place at least once a year, which will allow us to determine the rate of lesion development and estimate prognosis. Nevertheless, individuals with large, non-restrictive defects at the time of the first examination may already present with symptoms of heart failure, which would require immediate surgical intervention. In such cases, without appropriate surgical care, the long-term prognosis is made cautiously, and can be difficult to estimate.

The nomenclature of VSD, which is still used interchangeably nowadays, depending on the preference of the examiner, remains an important issue. However, the standardization of the nomenclature considering the latest divisions implemented by the ISNPCHD describes the location of the defects in relation to their topographical nature in the RV. They also indicate the physiological–electrical nature of VSDs concerning the heart’s electrical conduction. The four categories of VSD, namely, perimembranous central VSD, inlet VSD with common AV junction, trabecular muscular VSD, and outlet VSD, enable the precise determination of its location, significantly facilitating the cardiac surgeon’s assessment before possible surgery. For this reason, we also believe that standardizing and operating with a consistent, state-of-the-art nomenclature will facilitate communication among cardiologists in veterinary medicine, so we recommend that it be introduced into daily practice.

## Figures and Tables

**Table 1:** Presentation of the historical record of VSD classifications along with a description of their locations.

Approach	Author	VSD Classification		Description
Geographic	[74]	Related to ventricular outflow tracts		Ventricular septal defect is located anteriorly from the supraventricular crest involving the fibrous annulus region of the pulmonary valve and the aortic valve. The second is located posterior to the supraventricular crest involving the membranous portion of the interventricular septum and adjacent septal muscular portion
		Not related to ventricular outflow tracts		Ventricular septal defect associated with the muscular portion of the inflow ventricular septum and defects not associated with any of the atrioventricular valves
	[75]	Located above supraventricular crest—“supracristal”		Ventricular septal defect located above the supraventricular crest, i.e., in the ventricular outflow tract
		Located beneath supraventricular crest—“infracristal”		Ventricular septal defect located below the supraventricular crest, i.e., within the inflow tract of the ventricle
		Multiple muscular		Ventricular septal defect involving different localizations of the muscular portion of the interventricular septum
	[76]	Membranous septum	Membranous	Ventricular septal defect around the membranous part of the interventricular septum, often extended into the adjacent myocardium. Cradled by the limbs of a septal band and roofed by an infundibular septum. Malalignment between infundibular and ventricular septa with overriding of at least one semilunar valve may occur
			Perimembranous	
			Paramembranous	
		Inlet and trabecular	Muscular	Ventricular septal defect involves the basal part of the interventricular septum at the inflow region. Its posterior surface is the fibrous junction between the mitral and tricuspid valves. It may be associated with lateral or rotational (or both) malalignment between atrial and ventricular septa. Malalignment may be associated with overriding or straddling (or both) of either atrioventricular valve
			Muscular inlet	
			Muscular trabecular	
		Infundibular septum	Infundibular	Ventricular septal defect in the region of the infundibular septum with borders completely muscular or partially limited by the leaflets of the semilunar valves.
			Outlet	
			Supraristal	
	[77]	Atrioventricular canal type		The septum of atrioventricular canal is completely absent with straddling of the septal leaflet of the tricuspid valve.
		Muscular		Openings in the muscular ventricular septum, above and below the septal band
		Conoventricular		Ventricular septal defect lying between the conal septum and the ventricular septum. Partially encompassing the membranous portion of the ventricular septum and extending to the infundibular septum
		Conal		Ventricular septal defect just at the orifice of the great vessels
Borders	[78]	Perimembranous	Inlet	Ventricular septal defect in the portion of the membranous part with extension to the inflow region
			Trabecular	Ventricular septal defect in the portion of the membranous part with extension towards the apex of the heart
			Infundibular	Ventricular septal defect in the portion of the membranous part with extension towards the infundibulum and prolapse (overriding) of the non-coronary leaflet of the aortic valve
		Muscular	Posterior (inlet)	Ventricular septal defect below the septal leaflet of the tricuspid valve but with entirely muscular rims
			Trabecular	Ventricular septal defect around septal–marginal trabeculae, usually multiple
			Infundibular	Ventricular septal defect opening into outflow tract with completely muscular rims without prolapsing non-coronary valve leaflet of the aorta
		Subarterial infundibular		Ventricular septal defect in the infundibulum septal area where the aortic and pulmonary artery valves formed its upper border
	[79]	Conoventricular		Ventricular septal defect in the area of the mid portion of right ventricle and outlet portion of left ventricle. They can be juxtatricuspid, juxtamitral, or juxtaaortic. They may also be associated with the membranous part of the septum. Sometimes there are posteriori with leftward malalignment of the conal septum
		In RV outlet		Ventricular septal defect in the outflow portion area of left ventricle and right ventricle. They can be juxtapulmonary, juxtaaortic (with possible aortic valve leaflet prolapse), juxtaarterial or with completely muscular borders
		Inlet septal		Ventricular septal defect in the area of the inflow portion to the ventricle. They could be juxtatricuspid or juxtamitral, associated with the membranous part of the interventricular septum or with completely muscular rims. Sometimes associated with malalignment of the atrial to ventricular septum
		Trabecular		Ventricular septal defect in the anterior, middle or apical region
Hybrid	[73]	Type 1—subarterial, supracristal, conal, infundibular		Ventricular septal defect in the ventricular outflow tract area or conal septum below the semilunar valves
		Type 2—perimembranous, paramembranous, conoventricular		Ventricular septal defect associated with the membranous part of the septum bounded by atrioventricular valves, excluding cases classified as type 3
		Type 3—inlet, AV canal type		Ventricular septal defect of the inflow portion to the right ventricle, located just below the atrioventricular valve apparatus
		Type 4—Muscular		Ventricular septal defect with completely muscular borders unrelated to previous types. They may be multiple.

## Data Availability

No new data were created or analyzed in this study.

## Abbreviations

The following abbreviations are used in this manuscript:

Ao	aorta
AI	aortic insufficiency
ASD	atrial septal defect
AV	atrioventricular
AVSD	atrioventricular septal defect
ECG	electrocardiography
CHD	congenital heart defect
DCRV	double-chambered right ventricle
ISNPCHD	International Society for Nomenclature of Paediatric and Congenital Heart Disease
LV	left ventricle
LVOT	left ventricular outflow tract
PDA	patent ductus arteriosus
PS	pulmonic stenosis
Qp:Qs	pulmonary (Qp) to systemic flow (Qs) ratio
RBBB	right bundle branch block
RV	right ventricle
RVOT	right ventricular outflow tract
SAS	subaortic stenosis
VSD	ventricular septal defect
VSD:Ao	ventricular septal defect diameter to Aortic diameter ratio
Δp	pressure gradient
